# TAIM: Tool for Analyzing Root Images to Calculate the Infection Rate of Arbuscular Mycorrhizal Fungi

**DOI:** 10.3389/fpls.2022.881382

**Published:** 2022-05-03

**Authors:** Kaoru Muta, Shiho Takata, Yuzuko Utsumi, Atsushi Matsumura, Masakazu Iwamura, Koichi Kise

**Affiliations:** ^1^Graduate School of Engineering, Osaka Prefecture University, Osaka, Japan; ^2^Graduate School of Life and Environmental Sciences, Osaka Prefecture University, Osaka, Japan

**Keywords:** arbuscular mycorrhizal fungi, magnified intersections method, computer vision, pattern recognition, deep convolutional neural networks, system development

## Abstract

Arbuscular mycorrhizal fungi (AMF) infect plant roots and are hypothesized to improve plant growth. Recently, AMF is now available for axenic culture. Therefore, AMF is expected to be used as a microbial fertilizer. To evaluate the usefulness of AMF as a microbial fertilizer, we need to investigate the relationship between the degree of root colonization of AMF and plant growth. The method popularly used for calculation of the degree of root colonization, termed the magnified intersections method, is performed manually and is too labor-intensive to enable an extensive survey to be undertaken. Therefore, we automated the magnified intersections method by developing an application named “Tool for Analyzing root images to calculate the Infection rate of arbuscular Mycorrhizal fungi: TAIM.” TAIM is a web-based application that calculates the degree of AMF colonization from images using automated computer vision and pattern recognition techniques. Experimental results showed that TAIM correctly detected sampling areas for calculation of the degree of infection and classified the sampling areas with 87.4% accuracy. TAIM is publicly accessible at http://taim.imlab.jp/.

## 1. Introduction

Arbuscular mycorrhizal fungi (AMF) infect plant roots and are considered to improve plant growth (Treseder, 2013). Recent research (Kameoka et al., 2019) has succeeded in the axenic culture of AMF. Therefore, AMF may be mass-produced in the future and are predicted to be used as a microbial fertilizer. To evaluate the usefulness of AMF as a microbial fertilizer, we need to investigate the relationship between the degree of root colonization of AMF and plant growth.

The most commonly studied effect of AMF infections on plant roots is the absorption of phosphorus. However, some studies have shown that injecting mycorrhizal fungi was one of the causes of promoting phosphorus absorption (Van Der Heijden et al., 1998; Smith and Read, 2010; Richardson et al., 2011; Yang et al., 2012), while others have ruled it out (Smith et al., 2004). Therefore, as the results are still controversial, further research on the relationship between phosphorus and AMF is needed. To promote the research, objective evaluation of experiments conducted by different observers under different conditions is indispensable. Therefore, calculating a reliable AMF colonization degree is essential for the research.

In general, the degree of AMF colonization is calculated using the magnified intersections (MI) method (McGonigle et al., 1990). All steps of the MI method are performed manually and thus the method is extremely labor-intensive. Moreover, given that the colonization degree is assessed manually, the decision criterion and results will vary among observers. For these reasons, conducting a comprehensive survey with this method is difficult, and the relationship between AMF colonization and plant growth remains unclear. Clarification of this relationship requires a fixed criterion for estimation of AMF colonization and automation of estimation of colonization degree.

In this article we propose a method for automation of the MI method for estimation of AMF infection degree. Based on the proposed method, we developed an application system named “Tool for Analyzing root images to calculate the Infection rate of arbuscular Mycorrhizal fungi” (TAIM) (Muta et al., 2020). TAIM is a web-based application that automatically calculates the AMF colonization degree from microscopic images with 40x magnification prepared for an AMF infection rate measurement method. Using a machine-learning-based classifier, TAIM calculates the AMF infection rate objectively, unlike manual calculation. Moreover, TAIM has two functions that allow the user to be incorporated to boost its estimation accuracy. One is to upload their own data, which increases training data for TAIM. The other is to correct wrong estimation results, which improves the quality of training data. By retraining TAIM using the updated training data, the estimation accuracy of TAIM can be boosted. Experiments to evaluate the performance of the proposed method demonstrated that the sampling areas for calculation of the infection rate were detected correctly and the degree of infection was determined with 87.7% accuracy.

## 2. Related Work

As microscopic images are captured under stable lighting, the images are especially suitable for image processing. Therefore, many processing methods have been proposed.

The most popular target for processing of microscopic images is a cell. In many cases, images include too many cells for manual observation. Therefore, image processing methods for cell image analysis have been proposed as an alternative to human observation. To date, procedures for detection (Al-Kofahi et al., 2010; Buggenthin et al., 2013; Schmidt et al., 2018; Weigert et al., 2020), tracking (Debeir et al., 2005; Chen et al., 2006; Dzyubachyk et al., 2010), and cell counting (Lempitsky and Zisserman, 2010) have been proposed. Given the stable lighting used for observation of microscopic images, many methods previously proved to be relatively accurate even before the emergence of deep neural networks (DNNs). Subsequent to the advent of DNNs, the accuracy of detection and tracking has drastically improved (Xie et al., 2018; Korfhage et al., 2020; Kushwaha et al., 2020; Nishimura et al., 2020; Liu et al., 2021). In addition, methods for performing more challenging tasks, such as detection of mitosis (Su et al., 2017), three-dimensional cell segmentation (Weigert et al., 2020), nuclei (Xing et al., 2019), and chromosomes (Sharma et al., 2017) have been published.

Image processing is also used for microscopic medical images. It is practical to use microscopic images to diagnose a disease caused by abnormal cell growth, such as cancer. Many methods have been developed to detect cancer (Yu et al., 2016; Vu et al., 2019) and diagnose cancer from microscopic images (Song et al., 2017; Huttunen et al., 2018; Kurmi et al., 2020). Microscopic images are also helpful to detect infectious diseases. Malaria is an infectious disease for which image processing is the most widely used detection method, and various methods have been proposed for its detection and diagnosis (Ave et al., 2017; Muthu and Angeline Kirubha, 2020). In addition, virus detection methods (Devan et al., 2019; Xiao et al., 2021) have been proposed. For medical applications other than disease diagnosis, methods such as blood cell identification have been developed (Razzak and Naz, 2017).

A typical example of microscopic image analysis in plants is the analysis of pollen. As pollen grains are small, microscopic observation is essential. For example, pollen detection and recognition methods from air samples have been proposed (Rodrìuez-Damián et al., 2006; Landsmeer et al., 2009). Recently, a method applying DNNs has been developed (Gallardo-Caballero et al., 2019). Pollen is also an object of study in paleontology as well as in botany. Pollen analysis, or palynology, involves the study of pollen grains in fossil-bearing matrices or sediments for consideration of the history of plants and climatic changes, for example. As pollen classification requires a broad range of knowledge and is labor-intensive, methods for automated pollen classification (Battiato et al., 2020; Bourel et al., 2020; Romero et al., 2020) have been proposed to replace manual observation.

Recently, Evangelisti et al. (2021) developed AMFinder to analyze plant roots using deep learning-based image processing. This tool can detect AMF and visualize the degree of AMF colonization. These authors' motivation and methodology were similar to our own. The main differences between AMFinder and TAIM are as follows. TAIM is based on the MI method (McGonigle et al., 1990), which uses the intersections of grid lines to quantify AMF colonization of roots, whereas AMFinder divides an image into squares of a user-defined size. TAIM is designed to be a web-based application accessible to all users who can use a web browser, whereas AMFinder is a standalone application consisting of a command-line tool and a graphical interface that requires installation on a computer equipped with graphics processing units (GPUs) and users must set up the environment themselves.

## 3. Materials and Methods

### 3.1. Materials

The roots used for the dataset were from soybean. The soybean plants were grown in a glasshouse under an average temperature of 30.9°C in the experimental field of Osaka Prefecture University. Each plant was grown in a pot in sterilized soil and was subsequently inoculated with AMF (*Rhizophagus irregularis* MAFF520059). Therefore, AMF were the only fungi present in the soil of the pots. Table 1 lists the cultivar, place of origin, and number of days growth for the soybeans.

**Table 1:** Details of the soybean root dataset used in the experiment.

**Genotypes**	**Place of origin**	**Growing days**
M 581	India	48
OUDU	Korea	36
JAVA 7	Indonesia	52
U 1290-1	Nepal	54
KARASUMAME	Taiwan	44
KADI BHATTO	Nepal	59

The plants were removed from the pots and the roots were washed with water. The roots were softened using 10% potassium hydroxide to aid decolorization. We used 0.5% hydrogen peroxide aqueous solution to clear the softened roots and hydrochloric acid to neutralize. The roots were stained using 0.05% trypan blue to stain the AMF blue. The stained root sample is placed on a glass microscope slide with 0.25-mm-wide grid lines at 1.00 mm intervals. We use the glass microscope slide to make observation more efficient.

The prepared slides were observed under a 40x objective with an optical microscope (OLYMPUS BH-2) and images were captured with a digital camera (OLYMPUS PEN E-PLI). The resolution of the images was 4,032 × 3,024 pixels.

### 3.2. AMF Infection Rate Measurement Method

We automate a method to measure the infection rate of AMF based on the MI method (McGonigle et al., 1990), which is a popular and accurate method for estimation of AMF colonization degree (Sun and Tang, 2012). The MI method is an improved version of the grid-line intersect (GLI) method (Giovannetti and Mosse, 1980). The GLI method uses a dissecting microscope and measures the colonization degree as the ratio of colonized sampling areas. The MI method uses a light microscope, which provides higher resolution and therefore MI can measure the colonization degree more accurately.

The method we automated uses roots prepared as described in Section 3.1. An observer categorizes the roots at the intersections of the grid lines into four categories. In the MI method categorizes the sampling areas into four categories: negative, arbuscules, vesicles, and hyphae only. While the proposed method and MI method essentially perform the same procedure, there is one difference in the categorization of sampling areas between the methods: the MI method excludes the sampling areas that do not include roots in advance, whereas the proposed method does not. Therefore, we added a new class, “no root,” for the intersections that do not include roots, to enable the proposed method to classify the root-less sampling areas.

**Figure 1:**
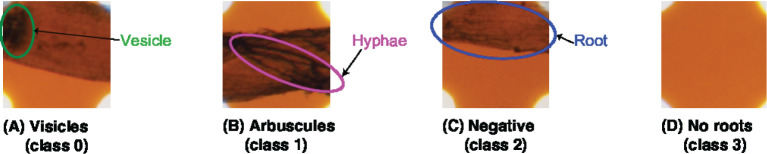
Target classes for the categorization. TAIM categorizes the sampling areas into four classes: **(A)** vesicles (class 0), **(B)** arbuscules (class 1), **(C)** negative (class 2), and **(D)** no root (class 3). Note that the four classes are not identical to those used in the MI method and the magnification of the slide images was 40x.

In addition, in our experiments, we integrated the “hyphae only” class into the “arbuscules” class to reflect the experimental environment. In our experiments, we sterilized the soil and then inoculated it with AMF. To avoid mixing the other microorganisms with the unsterilized soils in the pots, the plants were placed in a separate area from the unsterilized potted plants and treated to prevent soil contamination by watering. Hence, all the hyphae in the soil originate from AMF. In summary, we added a new class named “no root” and treated “hyphae only” as “arbuscules.” Therefore, in our experiments, we classified the sampling area into four categories: “vesicles,” “arbuscules,” “no root,” and “negative.” The original MI method uses a magnification of 200x, but this paper uses a 40x image. The reason for this is that 40x was sufficient for classifying in this study. Figure 1 shows a sample of each class.

After classifying the intersections, the proportions of arbuscular colonization (AC), vesicular colonization (VC) are calculated as


(1) $$\begin{array}{r} \text{AC}=\frac{N_{a}}{N_{s}-N_{\text{nr}}},\;\text{VC}=\frac{N_{v}}{N_{s}-N_{\text{nr}}}, \end{array}$$

where *N*_*a*_, *N*_*v*_, *N*_*n*_, and *N*_nr_ are the numbers of intersections categorized into arbuscules, vesicles, and no root, respectively, and *N*_*s*_ is the total number of intersections.

### 3.3. Software Design

We propose TAIM, a web-based application system to automate calculation of the degree of AMF colonization. In this section, we explain the system architecture of TAIM in Section 3.3.1, the method by which TAIM automatically calculates the infection rate of AMF in Section 3.3.2. We also explain a dataset we used for constructing TAIM in Section 3.3.3, and functions of TAIM in Section 3.3.4.

#### 3.3.1. Overview of TAIM

**Figure 2:**
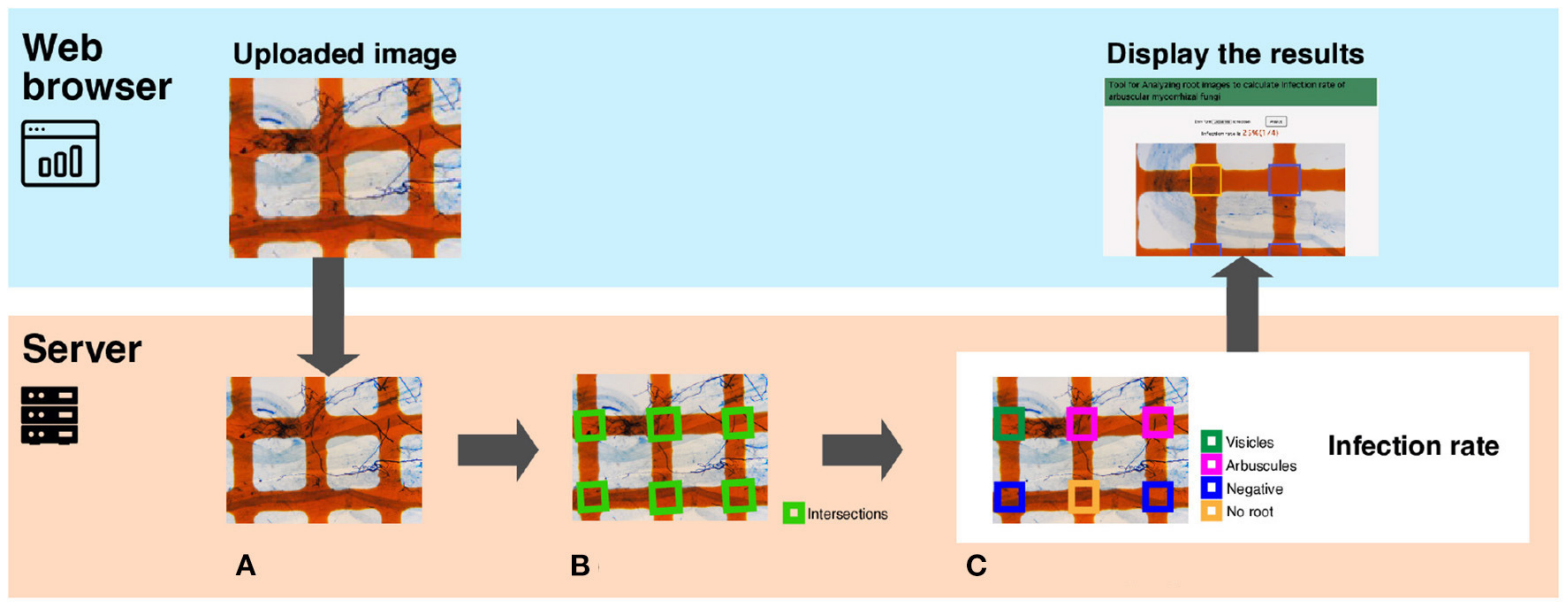
A system overview of TAIM. The system consists of a client and a server. The client, implemented on a web browser, receives the microscope slide images with the grid filter from users and shows the calculation results of the AMF colonization degree. The server receives images, calculates the AMF colonization degree, and returns the calculation results to the web client. The process for calculating the AMF colonization degree by the server are following; **(A)** The input image is a microscope slide image with a grid filter for calculating AMF colonization. **(B)** The intersections denoted by green rectangles are detected. They are used as sampling areas for calculating the AMF colonization degree. **(C)** Categorization results are visualized with different colors. The magnification of the slide images was 40x.

Figure 2 presents an overview of TAIM. TAIM consists of client and server systems. The client system runs on a web platform, receives images from users, and shows the calculation results. We use a web platform because it is independent of an OS environment and thus users can use TAIM on any device that can run a web browser. The server receives images from the client, calculates the AMF colonization degree, and transmits the results to the user. The server calculates the AMF colonization degree by detecting the sampling area using computer vision techniques and categorizing the sampling areas as colonized or not using machine-learning techniques. We use HTML5, CSS3, and JavaScript for client development and Django, which is a web application framework implemented by Python, for server development.

#### 3.3.2. Calculation of the AMF Colonization Degree

In this section, we explain the method for calculating the AMF colonization degree in detail. TAIM automates the method described in Section 3.2. Server part of Figure 2 shows an overview of the procedure for calculating the AMF colonization degree. The inputs are images captured from a microscope slide prepared for the MI method (Figure 2A). TAIM detects intersections of the grid lines as sampling areas for calculating the colonization degree. Examples of the intersections are shown as green rectangles in Figure 2B. TAIM then categorizes the sampling areas into four categories (Figure 1). Finally, TAIM calculates the AMF colonization degrees using Equation (1). Note that these four categories recognized by TAIM differ from the four categories of the MI method. Details are provided in Section 3.3.2.2.

##### 3.3.2.1. Intersection Detection

For intersection detection, we use a simple computer vision technique, namely edge detection using projection profile. The overall intersection detection process is shown in **Figure 4A**. The orange grid lines in the image are almost orthogonal (**Figure 4A**) If the lines are oriented in the horizontal or vertical direction of the image, the horizontal and vertical lines are expressed as the horizontal and vertical lines and are easily detected. Therefore, before intersection detection, we rotate the image so that the orange lines are oriented in the horizontal and vertical directions of the image. We term this process image rotation normalization.

**Figure 3:**
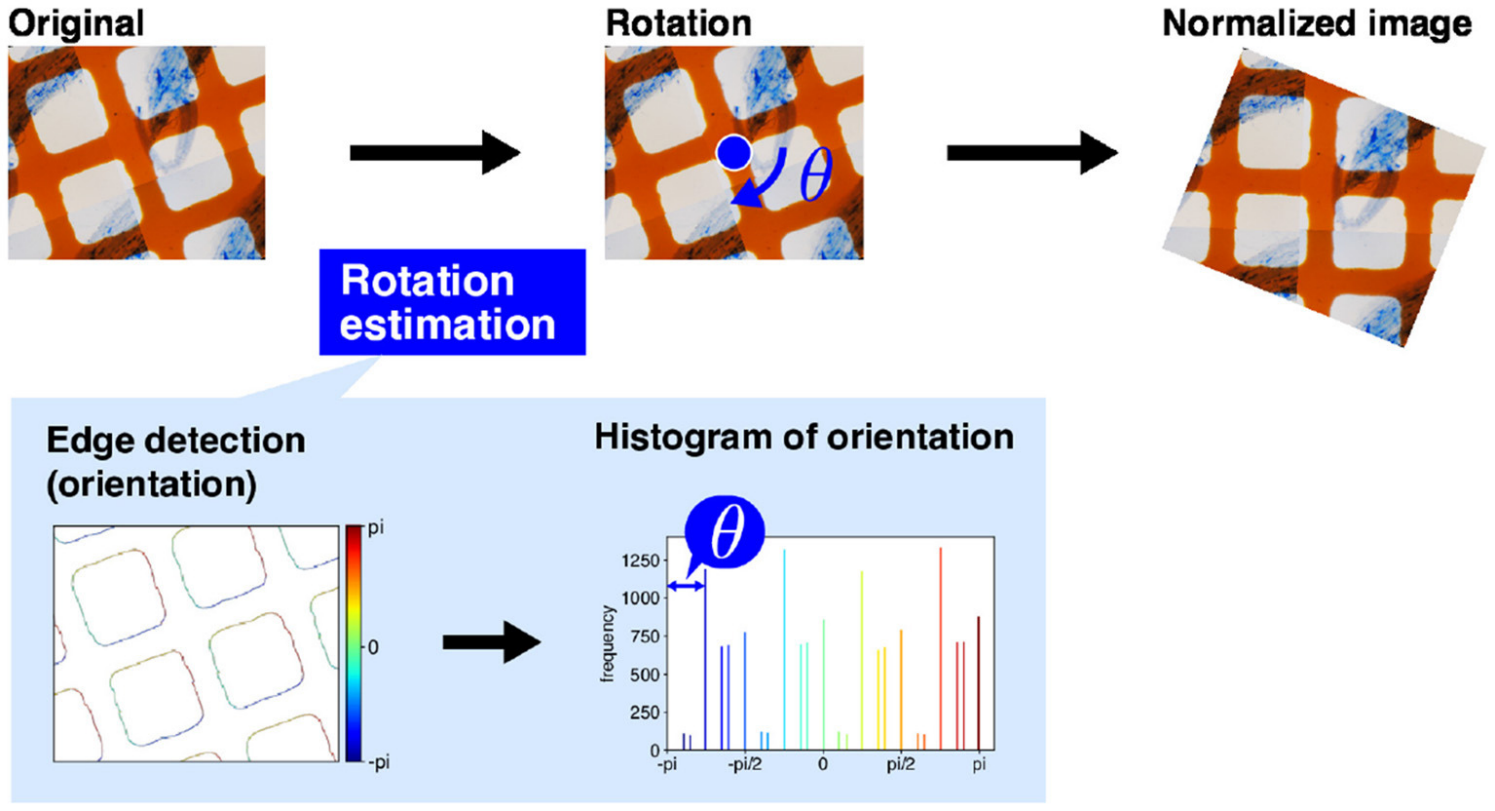
Image rotation normalization. First, the dominant angles in the original image are calculated to estimate the directions of the grid lines. The input image is then rotated. The magnification of the slide images was 40x. The blue arrow mark and blue circle dot are rotation direction of image normalization and image center, respectively.

Figure 3 shows the process of image rotation normalization. To execute image normalization, we estimate the angle of rotation. We use a histogram of the gradient directions of an input image to determine the angles. First, we apply a derivative filter, i.e., Sobel filter, horizontally and vertically to the input image to calculate the image gradients. We then calculate the gradient direction in each pixel and make a histogram of the gradient directions. As the orange lines are orthogonal, the histogram ideally has four peaks in every 90°. Therefore, the image should be rotated around the image center by the minimum angle that ensures the peaks are oriented in the horizontal and vertical directions. The image rotation normalization is applied to all input images.

**Figure 4:**
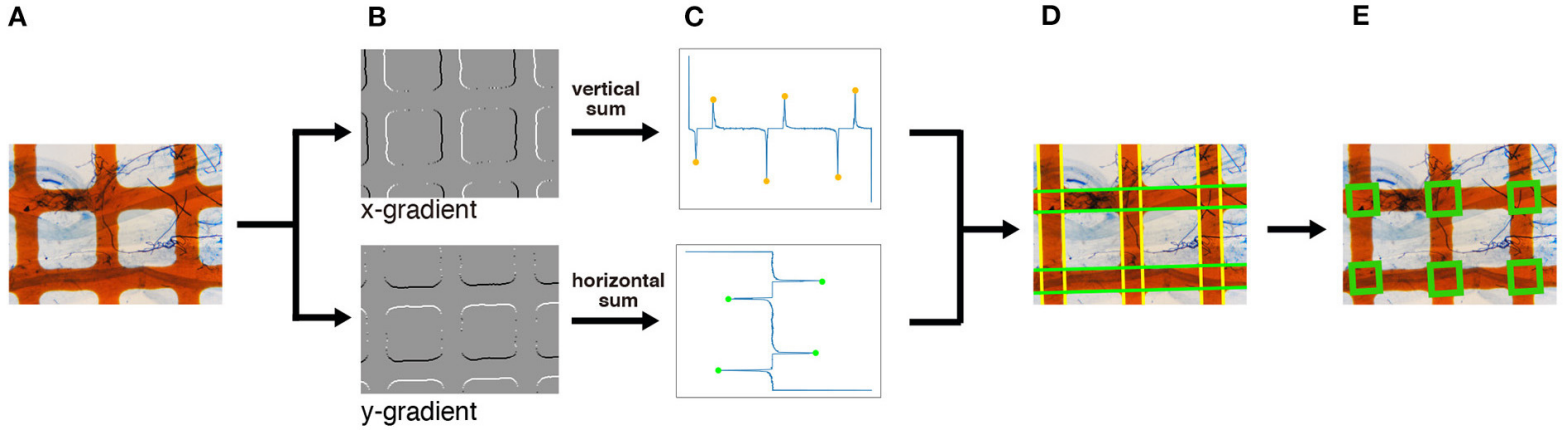
An overview of the intersection detection process. **(A)** Input image. The normalized image generated in Figure 3 is used as the input. **(B)** Edge detection. The *x*- and *y*-gradients are calculated by applying the Sobel filters horizontally and vertically, respectively. **(C)** Peak detection using projection profiles. The detected edges in **(B)** are projected horizontally and vertically, and the peaks are detected. **(D)** Grid line detection. Based on the peaks obtained in **(C)**, the horizontal and vertical grid lines, denoted in green and yellow, respectively, are detected. **(E)** Intersection detection. The intersections, denoted by green rectangles, are finally detected. The magnification of the slide images was 40x.

As shown in Figure 4, the intersections of the grid lines are detected on the normalized images in the following manner. We begin by applying the Sobel filter horizontally and vertically to the normalized image to calculate the gradient of each pixel (Figure 4B). We then detect the edges of the grid lines by detecting the peaks of the projection profiles of the gradients shown in Figure 4C. A projection profile is a sum of pixel values along an axis. Given that the grid lines in the normalized image are oriented in the horizontal and vertical directions, the edges of the lines appear as peaks of the horizontal and vertical projection profiles. As a result of the projection profile, we detect the edges of the grid lines, denoted in green in Figure 4D. The width of the grid lines is narrower than the distance between the lines (Figure 4A). Therefore, we regard a pair of green lines as a grid line if two green lines are at a close distance. Finally, we detect the intersections; the crossings of the grid lines are detected as intersections (Figure 4E).

##### 3.3.2.2. Categorization of the Sampling Areas

**Figure 5:**
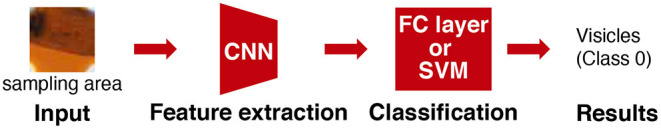
An overview of the classification process of the TAIM method. The magnification of the slide images was 40x.

We categorize the sampling areas (i.e., the intersections of grid lines) according to the degree of colonization using a pattern recognition technique. An overview of the categorization process is shown in Figure 5. We extract a feature vector from the input image (i.e., sampling area) and categorize it into four classes. In the feature extraction, we use convolutional neural networks (CNNs) because classifiers using CNNs have previously performed well in image classification tasks (Russakovsky et al., 2015; Krizhevsky et al., 2017). As good classification accuracy is expected when the feature is extracted by CNNs, we employ CNNs for feature extraction. We used CNN models pretrained on ImageNet (Deng et al., 2009), which consisted of more than 10 million images of 1,000 categories. After connecting a fully connected (FC) layer to the end of the pretrained CNN model, we fine-tuned the model in the task of categorizing the training images into four classes.

We used two kinds of classifiers in the categorization process: FC layer and support vector machine (SVM). In the former case, we used the FC layer that was used for pretraining. In the latter case, we used the SVM. To be more precise, we used the SVM for classification and the CNN model (without the FC layer) for feature extraction. In the training, the SVM was trained on the features extracted by the CNN model. Note that the SVM is a supervised learning model that shows good classification accuracy in biological image recognition (Noble, 2006). By using maximum-margin classifiers, the SVM achieves high recognition accuracy. Moreover, the SVM can cope with non-linear classification problems by introducing non-linear kernels.

The colonization degree is calculated as AC and VC in Equation (1) with at least 200 sampling areas.

#### 3.3.3. Dataset

We constructed an original dataset to construct TAIM and conduct experiments. The dataset consisted of 896 microscopic slide images prepared for as described in Section 3.1 (Figure 2A). We reduced the images to 1, 008 × 756 pixels for efficient detection and categorization of intersections. One of the authors manually classified the 5,002 intersection areas into four classes, which were mentioned in Section 3.2. We regarded the classification results as the ground truth (correct answers) of the data and used them to train and evaluate deep neural networks and SVM classifiers. Each intersection area was about 150 × 150 pixels.

#### 3.3.4. Functions

In addition to calculating AMF colonization degree, TAIM has two other functions: modifying the classification results by users and adding new data to improve the classification accuracy. These two functions are implemented to improve the classification accuracy and the objectivity of AMF colonization.

When viewing the classification results, as shown in Figure 2C), the modification function allows users to correct erroneous classification results. In pattern recognition, classifiers sometimes make mistakes depending on the lighting and changes in appearance because of the limitations of the training data. It is also true for the classifiers of TAIM. Therefore, we implemented the modification function to fix incorrect classification results.

TAIM also has a function to register new data, which is expected to improve the classification accuracy. Currently, the classifiers of TAIM are trained on only our dataset, which consists of soybean roots grown in the field and labeled by us. Therefore, the classifiers are expected to perform well on our data and on data with similar properties, but are under-learned for those with different properties. For the classifiers to be robust, a diverse dataset is essential. This is because root data grown in diverse locations are expected to help the classifiers of TAIM to acquire a strongly robust recognition capability. Hence, we implemented the function of TAIM that allows users to register new data that are used for additional training. Adding new data through the functions of TAIM, which stores the data and modifies the categorization results, is also expected to improve the objectivity of the categorization of colonization degree by TAIM. The initial TAIM classifiers for the colonization categorization are trained with the data labeled by one of the authors. That is, the trained classifiers of TAIM reflect the criteria of a single observer. The modified categorization results can reflect the criteria of other observers. Therefore, if the number of users involved in the labeling process increases, the classifiers reflect the criteria of multiple observers, leading to the classifiers becoming more objective.

## 4. Results

We conducted experiments to evaluate the detection and classification performance of TAIM using the dataset we created. This section presents details on the dataset as well as the detection and classification results.

### 4.1. Evaluation of Intersection Detection

We evaluated the intersection detection performance using the soybean root dataset. An overview of the proposed intersection detection method is presented in Section 3.3.2.1; here, we describe its implementation in more detail. We used the 3 × 3 Sobel filter as a derivative filter to calculate the gradient and direction of an image. From the image gradient direction, we estimated the rotation angle for normalization and normalized the image as described in Section 3.3.2.1. The projection profile used for line edge detection was calculated based on the gradient images. In the peak detection, we adopted a public domain code written in Python^1^. We set the distance parameter to 50 and used the default values for the other parameters to execute the code.

**Figure 6:**
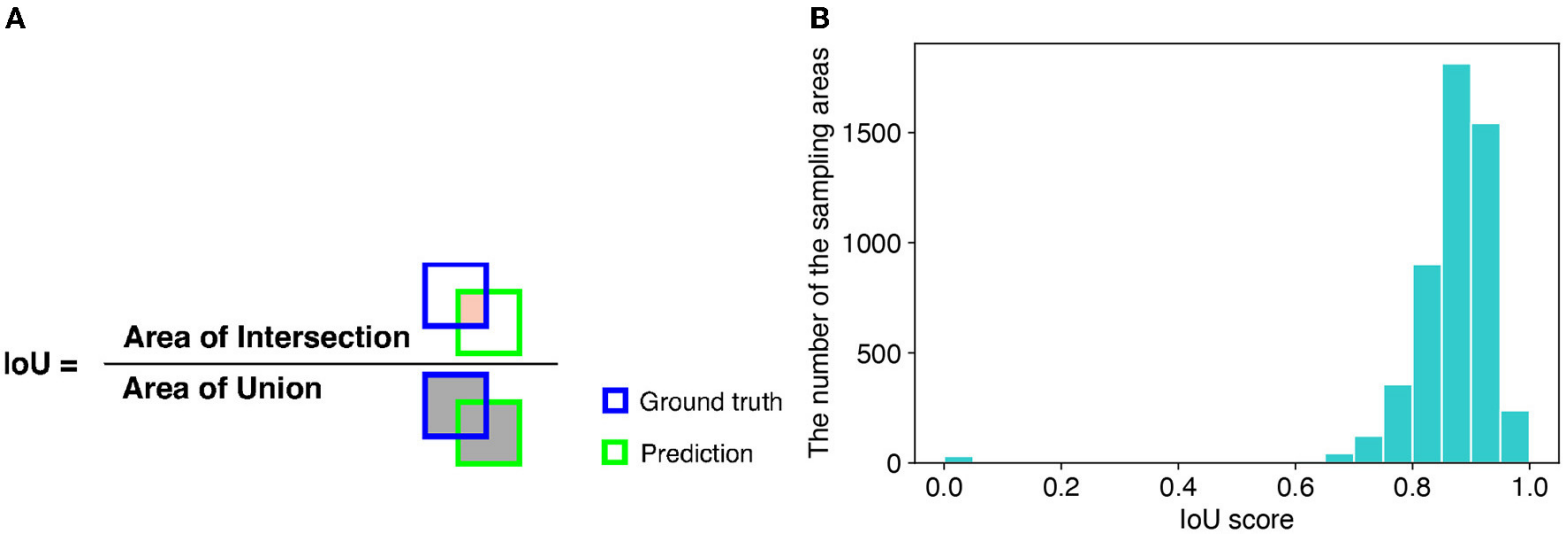
**(A)** Definition of the IoU. The IoU is calculated as the ratio of the intersection between the ground truth and predicted area, denoted in orange, over their union, denoted in gray. **(B)** Distribution of IoU scores in intersection detection.

For evaluation of detection performance, we adopted the intersection over union (IoU) score. The IoU is a criterion of how accurately a method detects the areas of target objects. The IoU is calculated as the ratio of the overlapping area (intersection) between the ground truth and predicted area over their union (Figure 6A). Therefore, the larger the IoU score, the more accurately the sampling areas are detected. We used 896 images of the soybean root dataset for the experiment to calculate the IoU scores. The mean IoU score of TAIM was 0.86. If we considered successful detection in the IoU score as more than 0.75, the detection precision of TAIM was 0.95. Therefore, TAIM achieved a satisfactory detection performance. Based on the distribution of the IoU scores (Figure 6B), most sampling areas were detected correctly; the IoU score of most sampling areas was >0.75. However, detection of a few sampling areas failed, as indicated by an IoU score close to zero.

To clarify the reason for the failure in detection, we compared examples of successful and failed detection. Supplementary Figure 1 shows examples of the detection experiments. The left column is an example of successful detection, and the central and right columns are examples of failed detection. In the detection results (the second row of Supplementary Figure 1), blue rectangles represent the ground truth and green rectangles the detection results. The IoU score of each detected area is shown in white text. In the peak detection results (the fifth and sixth rows of Supplementary Figure 1), detected peaks are indicated by red and green circles. In the left column, as the edges of the orange lines were clear, the edges were easily detected by peak detection. In contrast, in the central column, the edges of the orange lines were jagged. As a result, some sampling areas failed to be detected. In the right column, the input image contained bubbles, whereas the edges were clear. The bubble edges were incorrectly detected as a peak because the intensity of the gradient of the bubble edges was large.

### 4.2. Evaluation of Classification Accuracy

We conducted experiments to evaluate the classification accuracy of the proposed method. We used the cropped sampling areas from the soybean root dataset. We applied intersection detection, as described in Section 3.3.2.1, to the dataset and used the areas for which the IoU score was >0.5. The number of cropped sampling images was 5,002 and annotated manually. The images were cropped to be squares and normalized to 224 × 224 pixels using bilinear interpolation. The CNN models used for feature extraction were AlexNet (Krizhevsky et al., 2012), VGG-19 (Simonyan and Zisserman, 2015), and ResNet-18 (He et al., 2016). These models were pretrained on ImageNet and fine-tuned on the cropped sampling areas. The number of epochs, batch size, and initial learning coefficient were set to 20, 32, and 10e-4, respectively, and the learning rate was reduced by half every five epochs. For optimization, we used Adam with weight decay of 1e-5. For classification, we used the SVM with a radial basis function (RBF) kernel. The cost parameters of the SVM and RBF kernels were set to 1.0 and 0.25, respectively. The output from the network was compared with the annotation, and if they matched, the output was considered correct, and if they did not match, the output was considered incorrect. The percentage of correct outputs was considered to be the classification accuracy. We used five-fold cross-validation to evaluate the classification accuracy. We divided the cropped image samples into five subsamples. We fine-tuned the CNN models and trained classifiers with four subsamples and evaluated the remaining subsamples. We repeated the procedure five times so that all subsamples were evaluated, and the overall accuracy was averaged. Regardless of the combination of feature extractor and classifier, the classification accuracy was >84% (Table 2). The hightest classification accuracy (87.7%) was achieved when using VGG-19 as the feature extractor and FC as the classifier.

**Table 2:** Results of the classification experiment.

**Feature extractor (CNN)**	**Classifier**	**Accuracy (%)**
AlexNet	FC	84.1
VGG-19	FC	87.7
ResNet-18	FC	84.6
AlexNet	SVM	84.0
VGG-19	SVM	86.9
ResNet-18	SVM	84.9

**Figure 7:**
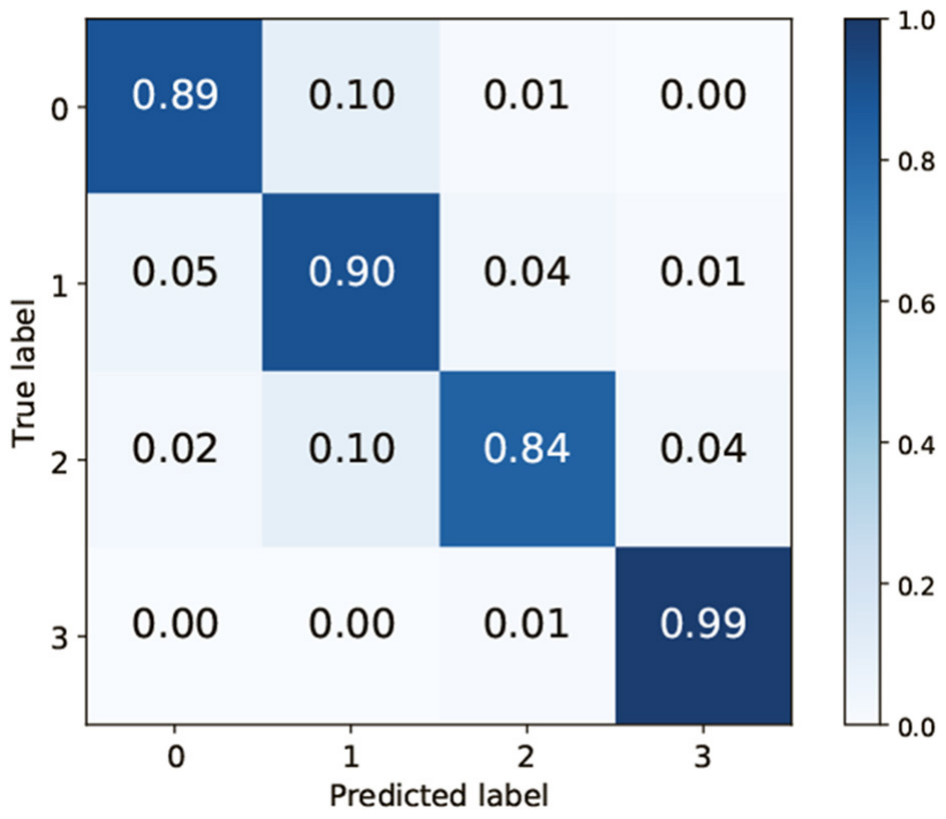
Confusion matrix when using VGG-19 as the convolutional neural network and the fully connected layer as the classifier.

To clarify which class was misrecognized, a confusion matrix when VGG-19 and FC were used for feature extraction and classification was generated. TAIM tended to confuse class 0 (vesicles) with class 1 (arbuscules) and class 2 (negative) with class 1 (Figure 7). There are two possible reasons for misclassification. First, the appearance of the respective classes was similar. Classes 0 (vesicles) and 1 (arbuscules) were similar, and classes 2 (negative) and 1 were also similar (Figure 1). Therefore, such similarity in appearance may have caused misclassification. The second possible reason is the imbalance of the training data. The number of training data for the four classes was 423, 1,351, 628, and 2,600, respectively. As classes with fewer training data tend to be treated as less important, samples belonging to classes 0 and 2 were more frequently misclassified compared with the other classes.

**Figure 8:**
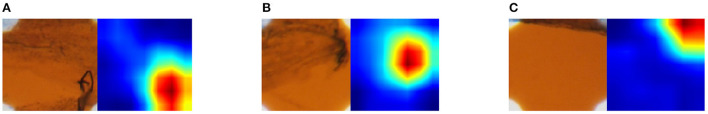
Original sampling area (left) and importance of sampling areas indicated by CAM (right). CAM visualizes the importance for classifying images. The more red the pixel values are, the more important for classification, and the more blue, the less important. The magnification of the slide images was 40x. **(A)** Classified an image of class 1 correctly. **(B)** Misclassified an image of class 1 as class 0. **(C)** Misclassified an image of class 1 as class 2.

We generated a class activation map (CAM) (Zhou et al., 2016) of the fine-tuned ResNet-18 to visualize how the CNN classified the sample. The CAM visualizes as a heat map the importance of regions for classification of the sample. In other words, important regions for classification of a class are considered to contain class-specific features for the class. Figure 8 shows three original images and the corresponding CAM; blue regions are more important and red are less important. Figure 8A is an example of an image that was classified as class 1 correctly. The CAM of this image showed that classification was based on the hyphae in the lower right corner. Figures 8B,C are examples of images that were misclassified; B was class 1 but classified as class 0, and C was class 1 but classified as class 2. The CAM of Figure 8B revealed that the only hyphae were located in the class-specific region. Therefore, the appearance of the original image in the region was similar to that of class 1. This appearance similarity led to the misclassification as the arbuscules class. In the original image of Figure 8C, hyphae were located at the top but only close to the edge of the image. Hence, this is a difficult sample to correctly classify. In the CAM, a class-specific area existed in the top right corner, which may exacerbate the misclassification.

**Figure 9:**
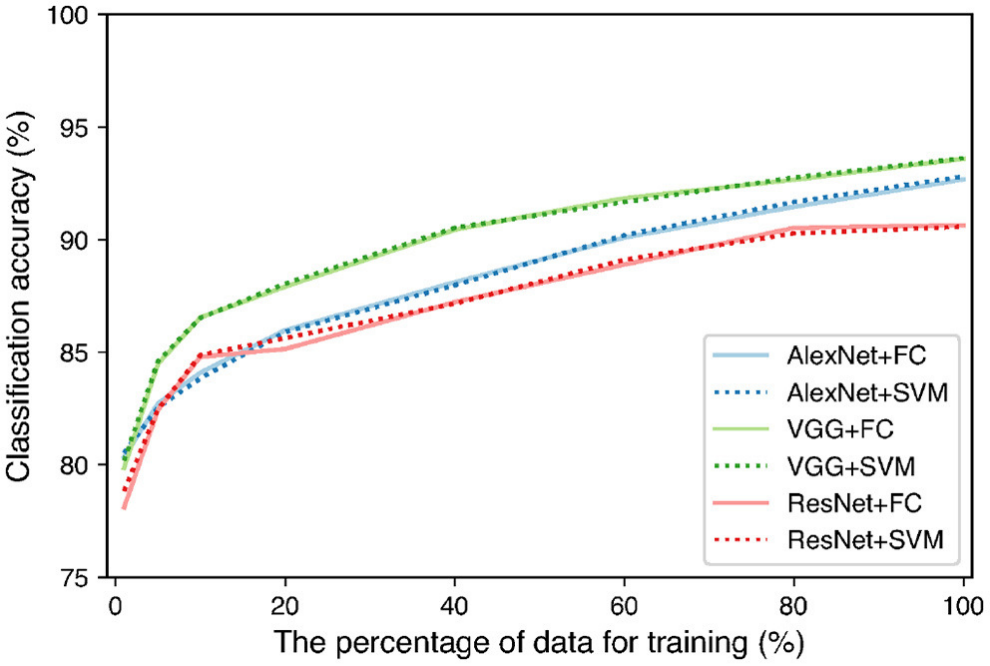
Classification accuracy with increase in percentage of training data.

As TAIM has a function that collects data from users, it is possible to increase the training data while TAIM is running. To clarify the effect of increasing the training data, we increased the number of images using augmentation techniques and observed the change in accuracy. We reflected images horizontally and vertically, cropped them randomly, and resized them to 224 × 224 pixels. The total dataset was increased to 300,012 images. The images were divided into training, validation, and test samples in the ratio of 3:1:1. We used the same feature extractors and classifiers as in Figure 2. We trained the networks while changing the training data from 1% of the total training data to 100%. We used the same hyperparameter setting for the networks as the previous classification experiment. We used validation data to evaluate the training accuracy of the networks and choose the parameter of the SVM. The training data were used for evaluation of the classification accuracy. Figure 9 shows the relation between the number of training data and the classification accuracy. The figure shows that all combinations between the networks and classifiers tended to increase the accuracy. In addition, the accuracy is expected to be further improved using more training data because the accuracy still showed an increasing trend when 100% data were used for training. Therefore, it is expected that the classification accuracy of TAIM will improve with increase in the amount of data used for training.

## 5. Discussion

In the experiments, we evaluated TAIM on soybean and one AMF. Further evaluation with other plants and AMFs should be conducted to demonstrate the usefulness of the proposed system.

AMFinder (Evangelisti et al., 2021), introduced in Section 2, is a method that shares a similar motivation with TAIM. The most significant difference of AMFinder from TAIM is the presence or absence of grids in the input microscope images. TAIM used microscope slide images with the grids, whereas AMFinder used those without the grids. Regarding the difference, we mention two points from the technical perspective. The first is that the classification algorithm for TAM and AMFinder is not inseparable but can be plug in. It means that when a better algorithm is developed in the future, we can improve the classification performance by plugging in to the methods. The second is that it is even easier to use slide images without grids than with grids. The images with grids can be treated as follows. Training data can be created by randomly cropping original slide images from images without grids and annotating them. Using the training data, the network can be trained to classify the cropped images without grids into the categories of AMF infection. Moreover, it is possible to identify which part of the image is infected even without grids using a sliding window approach, which crops images by sliding a rectangle of a fixed size on an image and classifying each cropped image. In our future work, we would like to extend TAIM so that infection rates can be calculated regardless of the presence or absence of grids in slide images.

Although TAIM can potentially distinguish hyphae and arbuscles following the MI method, due to the annotation effort and data limitations, we were unable to perform an experiment that distinguishes hyphae and arbuscles. In the future, when we obtain the appropriate data, we would be able to perform such an experiment. Similarly, the difference in the number of classes classified in TAIM and AMFinder comes from the difference in the data used in the experiments. TAIM classified infected roots into two classes (Arbuscules and Vesicles) in our experiments, whereas AMFinder did into four classes (Arbuscules, Vesicles, Hyphopodia, and Intraradical hyphae). This difference does not mean superiority or inferiority of the classifiers themselves but the difference in the data used in the experiments. Therefore, if we can collect the appropriate training data, of which class labels are the same as those of AMFinder, TAIM would be able to identify infected roots in the same detail.

Since annotation is done manually, errors are inevitable, and the error affects the classification accuracy. For example, apart from plat science, there is a well-used handwritten digit image dataset in the computer vision field, called MNIST dataset^2^. The test data of the MNIST dataset, which consists of 10,000 handwritten digit images, has 0.15% of labels that are expected to be wrong (Northcutt et al., 2021). Our dataset consists of a smaller number of images and fewer classes than MNIST. However, the annotation task of our dataset is harder than that of the MNIST dataset. Hence, it is difficult to expect the number of annotation errors in our dataset. However, even if there are errors, it would not seriously affect the classification accuracy insofar as the errors occur randomly. Actually, there are two types of errors that should be considered. One is annotation errors that occur randomly due to human error, which is argued above. The other is a bias that occurs depending on annotators, which is caused by having different criteria. The effect of the latter is more critical when fewer annotators are involved. Since TAIM is currently trained on data annotated by one of the authors, it is expected that the dataset is biased. However, when the number of annotators increases, it is expected that the bias will decrease. TAIM proves the function that decreases bias because TAIM can be used by multiple users and the users can upload their own data, as described in Section 3.3.4. Therefore, when the number of users increases, TAIM is expected to provide better classification results than those manually annotated by a single person.

## 6. Conclusion

This article describes a web-based application called TAIM, which calculates the colonization degree of AMF automatically from microscopic images. As AMF is now available for axenic culture, AMF is expected to be used a microbial fertilizer. To evaluate of the effectiveness of the AMF as a microbial fertilizer, colonization degree of AMF is required. One impediment to such research is that estimation of the colonization degree of AMF is still presently conducted manually. Therefore, we developed TAIM to automate calculation of the extent of AMF colonization. Because TAIM is a web-based application, it can be used *via* a web browser and does not require users to set up a calculation environment. TAIM also has a function to collect new training data from users and retrains the classifier of colonization. This function will contribute to reduction in variation of the decision criteria by observers by combining data annotated by multiple observers. We evaluated the detection and classification accuracy of TAIM with an experimental soybean root dataset comprising cropped 5,002 intersection areas. The experimental results showed that TAIM detected the intersection regions with a mean IoU score of 0.86. If an area with an IoU score of 0.75 is considered to represent successful detection, TAIM can detect the intersection regions with 95% accuracy. TAIM classified the detected regions into four classes with 87.7% accuracy. The classification accuracy improved with increase in number of training data. Therefore, the estimation accuracy of AMF colonization degree is predicted to improve by using the data collection function. TAIM is expected to contribute to an improved understanding of the effect of AMF as a microbial fertilizer.

## Data Availability Statement

The raw data supporting the conclusions of this article will be made available by the authors, without undue reservation.

## Author Contributions

YU conceptualized the research, managed the project, and sourced funding. KM, YU, and MI developed the methodology. KM and YU developed the software and wrote the original draft of the manuscript. KM performed the validation, formal analysis, and investigation. AM and KK prepared the materials for the research. ST and KM were involved in data curation. MI and AM reviewed the manuscript. All authors have read and agreed to the final version of the manuscript.

## Funding

This research was funded by the Osaka Prefecture University Female Researcher Support Project.

## Conflict of Interest

The authors declare that the research was conducted in the absence of any commercial or financial relationships that could be construed as a potential conflict of interest.

## Publisher's Note

All claims expressed in this article are solely those of the authors and do not necessarily represent those of their affiliated organizations, or those of the publisher, the editors and the reviewers. Any product that may be evaluated in this article, or claim that may be made by its manufacturer, is not guaranteed or endorsed by the publisher.

## Footnotes

^1^ https://gist.github.com/endolith/250860

^2^ http://yann.lecun.com/exdb/mnist/
