# Supplementary material for: TAIM: Tool for Analyzing Root Images to Calculate the Infection Rate of Arbuscular Mycorrhizal Fungi
Source: Front Plant Sci. 2022 May 3;13:881382. doi: 10.3389/fpls.2022.881382 (PMC9111841; doi:10.3389/fpls.2022.881382)
Supplement: Supplementary file 1 [file Data_Sheet_1.pdf]

frontiers

Supplementary Material

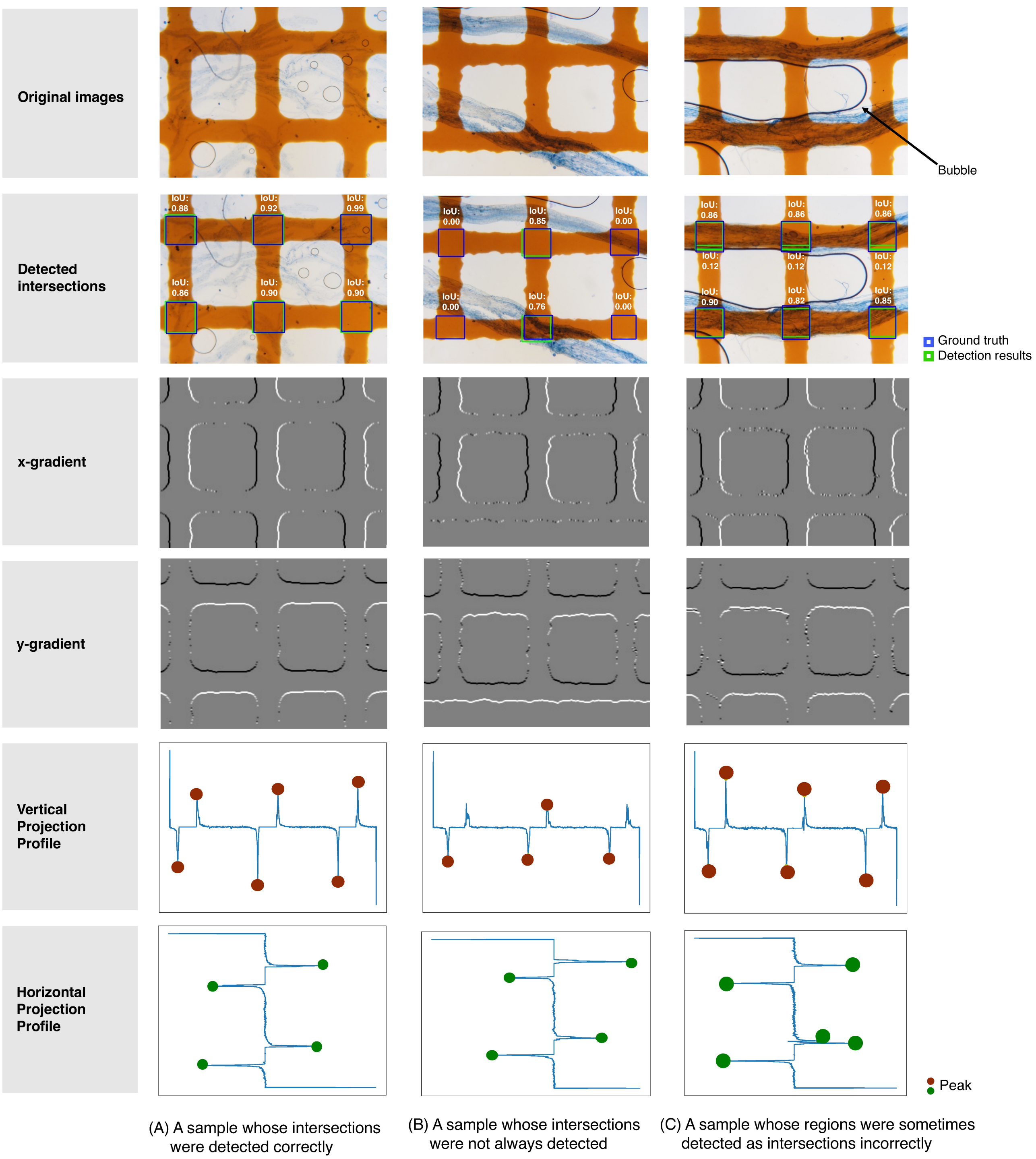

Figure S1. Examples of the results of the detection accuracy experiment. The first row shows the original images. The second row shows the detected intersections. The third and fourth rows show the $x-$ and $y-$ gradients of the images, respectively. The fifth and sixth rows show the vertical and horizontal projection profiles, respectively. The magnification of the slide images was 40x.
